# Boerhaave Syndrome Causing Bilateral Empyemas

**DOI:** 10.14309/crj.0000000000000203

**Published:** 2019-09-02

**Authors:** Divya Chalikonda, Joseph Yoo, Drew Johnson, Christina Tofani

**Affiliations:** 1 Department of Internal Medicine, Thomas Jefferson University Hospital, Philadelphia, PA; 2 Department of Internal Medicine, Division of Gastroenterology and Hepatology, Thomas Jefferson University Hospital, Philadelphia, PA; 3 Department of Internal Medicine, Division of Cardiology, Thomas Jefferson University Hospital, Philadelphia, PA

## Abstract

Boerhaave syndrome is a perforation of the esophagus caused by a sudden increase in intraluminal pressure. It is known to be associated with left-sided pleural effusion and mediastinitis, but rarely presents with bilateral effusion. Its association with the presence of a hiatal hernia is unclear. We present a patient with a hiatal hernia who developed bilateral empyemas because of Boerhaave syndrome and was treated with an endoscopically placed esophageal stent.

## INTRODUCTION

Boerhaave syndrome is a barogenic tear of the esophagus, typically at the gastroesophageal junction, caused by a sudden increase in intraluminal pressure in the distal esophagus. Classically, patients with Boerhaave syndrome present with chest pain, vomiting, and a unilateral left pleural effusion.^1,2^ Patients also may have mediastinal free air, subcutaneous emphysema, and mediastinitis.^2,3^ We describe a case of Boerhaave syndrome causing bilateral empyemas in a patient with a hiatal hernia who presented with nausea and vomiting secondary to diabetic ketoacidosis.

## CASE REPORT

**Figure 1.**
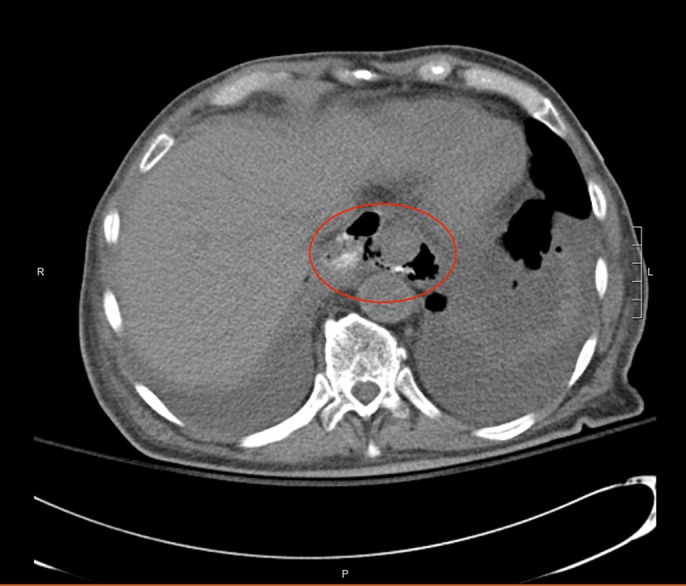
Chest computed tomography with oral and without intravenous contrast on admission. The circled area designates foci of extraluminal air adjacent to the distal esophagus tracking to the pleura or medial left lower lobe.

A 74-year-old man with type 2 diabetes mellitus and coronary artery disease was admitted to a nearby hospital with worsening mental status, epigastric abdominal pain, nausea, and emesis. Initial laboratory tests were consistent with diabetic ketoacidosis, and he was admitted for further management. His hospitalization was complicated by progressive hemodynamic instability and hypoxic respiratory failure requiring intubation. Abdominal and pelvic computed tomography (CT) with oral and without intravenous contrast demonstrated bilateral pleural effusions with bibasilar subsegmental consolidation and a large hiatal hernia with a possible small tract extending from the contrast-filled hiatal hernia to the pleura or lung of the medial left lower lobe (Figure 1).

A right chest tube was placed. Initial pleural fluid studies from the right hemithorax showed a white blood cell count of 13,711/mL^3^, protein of 2.1 mg/dL, and lactate dehydrogenase of 1,578 U/L. Cultures were positive for group D *Enterococcus faecalis*, methicillin-resistant *Staphylococcus aureus* (methicillin-resistant *S. aureus*), and yeast. Repeat pleural fluid studies 4 days later demonstrated a white blood cell count of 240,906/mL^3^ with the same organisms cultured. These results prompted concern for esophageal perforation causing bilateral empyema. He was transferred to our tertiary care center for further workup.

**Figure 2.**
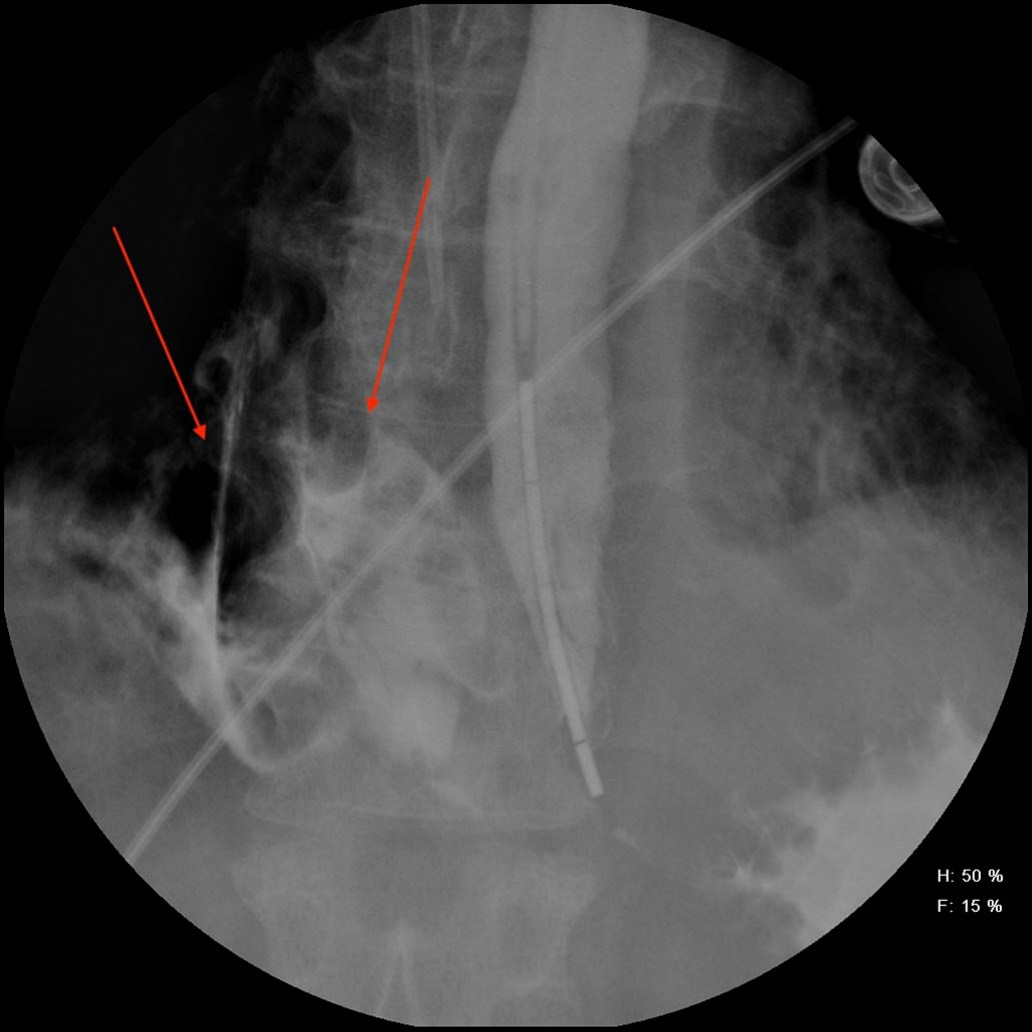
Contrast-filled enteric tube in the stomach with extravasation of contrast (arrows) evident at the distal esophagus in to the right pleural space.

**Figure 3.**
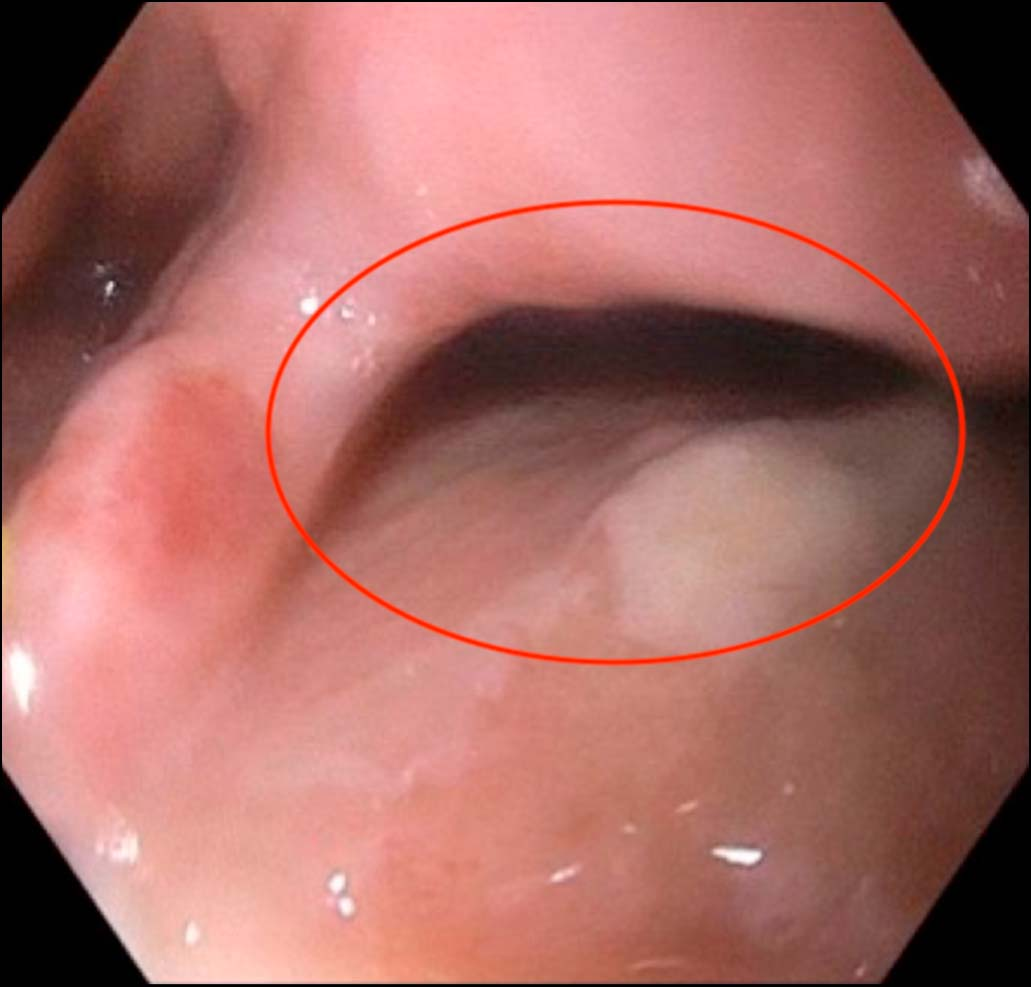
Endoscopic image of 1-cm esophageal wall defect identified 36 cm from the incisors.

On transfer, the patient remained intubated and sedated. Physical examination noted decreased breath sounds bilaterally at the lung bases. His right chest tube was in place to suction. He was diffusely edematous. No chest wall crepitus was present. The remainder of the examination was unremarkable. Repeat CT of the chest, abdomen, and pelvis without intravenous or oral contrast showed moderate bilateral pleural effusions without evidence of esophageal perforation. Additional chest tubes were placed bilaterally that drained pus. Pleural studies bilaterally were consistent with empyema, and cultures grew methicillin-resistant *S. aureus*, *E. faecalis*, *Eikenella corrodens*, and several other oral flora. Given the concern for esophageal rupture, he underwent a Gastrograffin swallow study, which demonstrated contrast extravasation into the mediastinum and right pleural space from the distal esophagus, consistent with esophageal perforation (Figure 2). The following day, he underwent esophagogastroduodenoscopy with placement of a fully covered 23 mm × 100 mm EndoMAXX esophageal stent (Merit Medical Endotek, South Jordan, UT; Figure 3).

The remainder of the patient's medical course was complicated by persistent right-sided empyema, ultimately requiring video-assisted thoracoscopic surgery with decortication. Upper endoscopy 2 months after the stent placement revealed a healed perforation, at which point the stent was removed. Currently, the patient is being slowly reintroduced to a mechanical soft diet with a tube-fed nutritional supplementation.

## DISCUSSION

Boerhaave syndrome's classic presentation, Meckler's triad (retching, chest pain, and subcutaneous emphysema), is present in less than 10% of cases, which frequently leads to a delayed or missed diagnosis.^1^ Clinical presentation varies by severity, although, if left untreated, it carries a high mortality rate. Therefore, it should be considered when any 2 of Meckler's triad are present in a patient's history.^1,2^

A Boerhaave perforation is classically localized to the left distal third of the esophagus, likely because of a weak zone in the smooth muscle fibers at this level.^3^ Owing to the usual location of perforation, 75%–90% of cases result in a unilateral left-sided pleural effusion that can progress to empyema.^4^ Although unilateral effusion is somewhat common with this pathology, only 1 case report has demonstrated bilateral empyemas as a consequence of Boerhaave syndrome.^5,6^ This patient's fluoroscopy only demonstrated a leak into the mediastinum and right pleural space, which is likely because of delayed imaging, allowing a left-sided tear to heal.

Our patient was found to have a hiatal hernia, which may lead one to question whether it predisposed to bilateral esophageal perforations. This association is not well studied; however, a review of 34 patients with Boerhaave syndrome found 8 with a hiatal hernia, indicating this pathology is of relatively increased frequency in patients with esophageal rupture.^7^ This idea is supported by the proposed theory that the presence or absence of a hiatal hernia can influence the location of a mucosal tear.^8^ Further study is warranted to better assess this relationship because the presence of a hiatal hernia could heighten suspicion for Boerhaave syndrome in a patient with an otherwise equivocal presentation.

Mortality rates from Boerhaave syndrome have improved compared with previous decades, given the advances in treatment and imaging.^9^ Fluoroscopic esophagography was standard for years, which has now changed to CT.^2,8,10^ The literature comparing these 2 modalities in diagnosing Boerhaave syndrome suggest a higher sensitivity and negative predictive value of CT in comparison with fluoroscopic esophagography.^11^ Studies have documented 100% sensitivity and 100% negative predictive value with CT compared with 73.3% and 95.7%, respectively, for fluoroscopic esophagography, for identifying esophageal perforation.^12,13^ Importantly, on multiple CTs, our patient did not have convincing evidence of pneumomediastinum but did have a definitive diagnosis made by a swallow study.^4,14^

Current treatment approaches for Boerhaave syndrome include conservative, endoscopic, or surgical management, with mortality rates documented as 75%, 100%, and 81%, respectively.^11^ Ongoing debates exist regarding best practices based on a patient's presenting clinical scenario, with the current literature suggesting that endoscopic therapy be reserved for early presenters, those with a defect less than 1 cm and without evidence of sepsis, thus indicating a promising role for endoscopy in a carefully selected population with Boerhaave syndrome.^15,16^

Boerhaave syndrome is a life-threatening condition with several modalities for diagnosis and treatment. Its presentation can be variable, but even in those with atypical preceding symptoms, it is important to keep in the differential because of varying sensitivities and predictive values of imaging tests. Multiple treatment options exist, but a minimally invasive endoscopic approach, perhaps with endoluminal stent placement, clip closure, or suturing, should be considered in the right patient population.

## DISCLOSURES

Author contributions: D. Chalikonda, J. Yoo, and D. Johnson wrote the manuscript. C. Tofani edited the manuscript. D. Chalikonda is the article guarantor.

Financial disclosure: None to report.

Informed consent was obtained for this case report.
